# Clinical significance of *Anoctamin-1* gene at 11q13 in the development and progression of head and neck squamous cell carcinomas

**DOI:** 10.1038/srep15698

**Published:** 2015-10-26

**Authors:** Juan P. Rodrigo, Sofía Tirados Menéndez, Francisco Hermida-Prado, Saúl Álvarez-Teijeiro, M. Ángeles Villaronga, Laura Alonso-Durán, Aitana Vallina, Pablo Martínez-Camblor, Aurora Astudillo, Carlos Suárez, Juana María García-Pedrero

**Affiliations:** 1 Servicio de Otorrinolaringología, Hospital Universitario Central de Asturias and Instituto Universitario de Oncología del Principado de Asturias, Oviedo, Spain; 2 Servicio de Anatomía Patológica, Hospital Universitario Central de Asturias and Instituto Universitario de Oncología del Principado de Asturias, Oviedo, Spain; 3 Bioestadística, Hospital Universitario Central de Asturias, Oviedo, Asturias, Spain; 4 Universidad Autónoma de Chile, Santiago, Chile

## Abstract

This study investigates the clinical significance of *Anoctamin-1* gene mapping at 11q13 amplicon in both the development and progression of head and neck squamous cell carcinomas (HNSCC). ANO1 protein expression was evaluated by immunohistochemistry in a cohort of 372 surgically treated HNSCC patients and also in 35 laryngeal precancerous lesions. *ANO1* gene amplification was determined by real-time PCR in all the laryngeal premalignancies and 60 of the HNSCCs, and molecular data correlated with clinical outcome. *ANO1* gene amplification was frequently detected in both premalignant lesions (63%) and HNSCC tumours (58%), whereas concomitant ANO1 expression occurred at a much lower frequency (20 and 22%). Interestingly, laryngeal dysplasias harbouring *ANO1* gene amplification showed a higher risk of malignant transformation (HR = 3.62; 95% CI 0.79–16.57; *P* = 0.097; Cox regression). ANO1 expression and gene amplification showed no significant associations with clinicopathological parameters in HNSCC. However, remarkably ANO1 expression differentially influenced patient survival depending on the tumour site. Collectively, this study provides original evidence demonstrating the distinctive impact of ANO1 expression on HNSCC prognosis depending on the tumour site.

Head and neck squamous cell carcinoma (HNSCC) is the sixth most common cancer worldwide. Despite major advancements in cancer diagnosis and treatment, the survival rate for patients with HNSCC has only marginally improved over the past few decades^1^. Our ability to prognosticate advanced cases of HNSCC is especially poor owing to variations in the biological behaviour of tumours and inadequacies of the present staging system. Hence, it is essential to identify new markers that can distinguish differences in tumour condition and augment the predictive power of the current clinical markers^2^.

Amplification of chromosomal region 11q13 has been described in various human carcinomas, including those of the head and neck, breast, oesophagus, lung, ovary and bladder (reviewed in^3^). This genetic alteration is one of the most frequently observed in HNSCC (20–52%), found to correlate with aggressive tumour growth, the presence of lymph node metastasis and poor prognosis^4^.

The core region of amplification in HNSCC has been identified, which contains multiple genes showing increased expression associated to gene amplification^5^. Furthermore, it has been recently demonstrated using high-resolution genomic and transcriptomic microarray analysis that the amplification and expression of various genes within the 11q13 amplicon may predict the risk of developing distant metastasis in HNSCC patients^6^. On this basis, Anoctamin-1 gene (ANO1, also termed TMEM16A, ORAOV2, DOG1, TAOS2 and FLJ10261), encoding a calcium-activated chloride channel, has emerged as a strong candidate to drive 11q13 amplification in HNSCC by providing growth advantage to tumours and favouring metastatic dissemination^7^.

These important observations prompted us to perform a comprehensive study to investigate the clinical significance of ANO1 expression and gene amplification in a large series of HNSCC tissue specimens to establish ANO1 role in patient prognosis and disease outcome. Furthermore, the present study explores for the first time ANO1 role in early stages of HNSCC tumourigenesis and malignant transformation by analysing both ANO1 protein expression and gene amplification in laryngeal premalignant lesions, and also the correlations with the risk of progression to laryngeal cancer.

## Results

### Analysis of ANO1 protein expression and gene amplification in HNSCC

**Figure 1:**
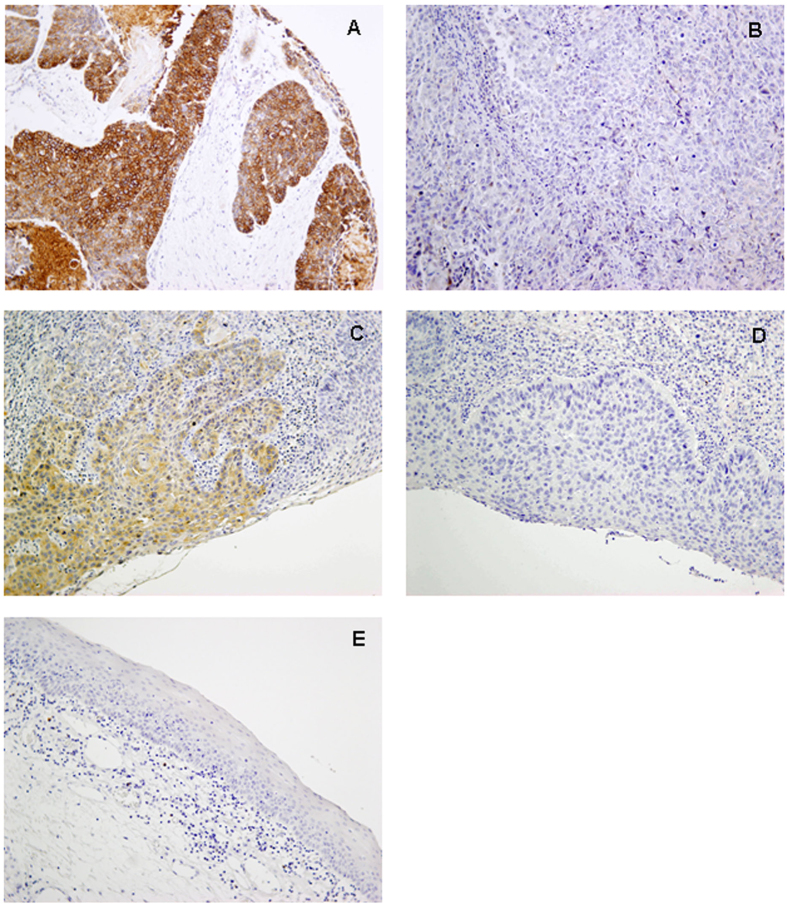
Immunohistochemical analysis of ANO1 protein expression. Representative examples of head and neck carcinomas showing strong ANO1 staining (**A**), negative staining (**B**), and laryngeal dysplasias with strong ANO1 staining (**C**) and negative staining (**D**). Normal adjacent epithelia showed negligible ANO1 staining (**E**). Original magnification x200.

Immunohistochemical analysis of ANO1 expression was carried out on HNSCC TMAs composed of tissue sections from 372 HNSCC. Each TMA also contained sections of normal epithelium as an internal control. Immunostaining was successfully evaluated in 357 (96%) of 372 cases. An overview of all clinicopathological data of these patients is given in Table 1. 78 (22%) of the 357 tumours exhibited positive ANO1 expression (Fig. 1A,B) showing preferentially a membranous pattern, whereas ANO1 expression was negligible in both normal epithelium and stromal cells (Fig. 1).

*ANO1* gene amplification was investigated in 60 HNSCC tissue specimens by real-time PCR (Q-PCR). *ANO1* gene amplification was detected in 35 (58%) tumour samples with relative copy numbers ranging from two- to 19-fold (median, 6.1 fold). Statistical analysis revealed a positive correlation between ANO1 protein expression and gene amplification (Spearman’s (rho) = 0.378, *P* = 0.003). All tumours showing ANO1-positive expression harboured *ANO1* gene amplification; however, gene amplification did not lead to increased expression in all cases.

### Associations with clinicopathological parameters and disease outcome

ANO1 protein expression was correlated with clinicopathological parameters (Table 1). ANO1-positive expression was found at each anatomic site, although with a higher frequency in oropharyngeal and hypopharyngeal tumours compared to laryngeal tumours (*P* = 0.065). No significant associations between ANO1 expression and other clinicopathological parameters were observed in our patient cohort (Table 1).

Nevertheless, ANO1 expression showed an impact on disease outcome. Multiple decrement Kaplan-Meier demonstrated that ANO1 expression is associated with increased disease-specific (DSS) and overall (OS) survival in the total cohort of 357 patients, although the differences did not reach statistical significance (Table 2).

To further confirm these results we repeated the immunohistochemical analysis of ANO1 expression using a different antibody (anti-TMEM16A antibody [SP31] from Abcam). 78 (22%) out of 350 cases showed positive ANO1 expression and a highly significant correlation was found when comparing ANO1 immunostaining detected with both antibodies (Spearman’s rho 0.917, P < 0.001). Furthermore, analogous findings were obtained regarding the impact of ANO1 expression (with [SP31] antibody) on patients’ survival (Supplementary Table S1).

Given that tumour site exerts an important influence on the prognosis of HNSCC patients, the impact of ANO1 expression on disease course was also examined separately in each anatomic site. Important differences were observed on patient survival between the different subgroups. Thus, patients with ANO1-positive oropharyngeal tumours exhibited a significantly improved DSS and OS (Table 2); whilst ANO1 expression showed no influence on survival of patients with hypopharyngeal, and was associated with a poorer survival, although not significant, in laryngeal tumours (Table 2). These results were confirmed by repeating ANO1 expression analysis with [SP31] antibody (Supplementary Table S1).

Amplification of *ANO1* gene did not show any significant association with clinical parameters or disease outcome. The 5-year DSS for non-amplified and amplified cases was 58% and 52% (HR = 1.15; 95% CI 0.60–2.2; *P* = 0.68), and the 5-year OS for non-amplified and amplified cases was 49% and 39% (HR = 1.2; 95% CI 0.67–2.14; *P* = 0.54).

### Analysis of ANO1 protein expression and gene amplification in early stages of tumourigenesis

ANO1 protein expression and gene amplification was evaluated on a set of 35 laryngeal premalignant lesions to determine their timing and frequency during HNSCC tumourigenesis.

**Figure 2:**
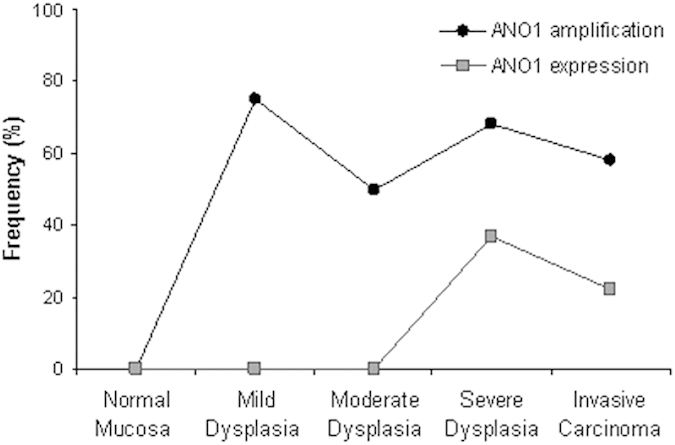
Frequencies of ANO1 protein expression and gene amplification in the different stages of laryngeal tumourigenesis.

Positive ANO1 expression was detected in 7 (20%) out of 35 laryngeal dysplasias (Fig. 1C,D), whilst *ANO1* gene amplification was found at a much higher frequency (63%, 22/35 laryngeal dysplasias). Clear differences were observed in relation to the histopathologic classification (Fig. 2). Thus, *ANO1* gene amplification occurred very early during tumourigenesis, thereby being detected in patients with mild dysplasias and at a high frequency along the different stages of tumour progression (Table 3). In contrast, ANO1 protein expression was only detected in severe dysplasia/carcinoma *in situ* (CIS) and at a much lower frequency than gene amplification (Table 3 and Fig. 2).

Statistical analysis revealed a significant correlation between ANO1 protein expression and gene amplification (Spearman’s (rho) = 0.401, *P* = 0.019). However, even though all seven ANO1-positive dysplasias harboured *ANO1* gene amplification, most cases with increased gene copy number showed ANO1-negative expression.

### Associations with laryngeal cancer risk

**Figure 3:**
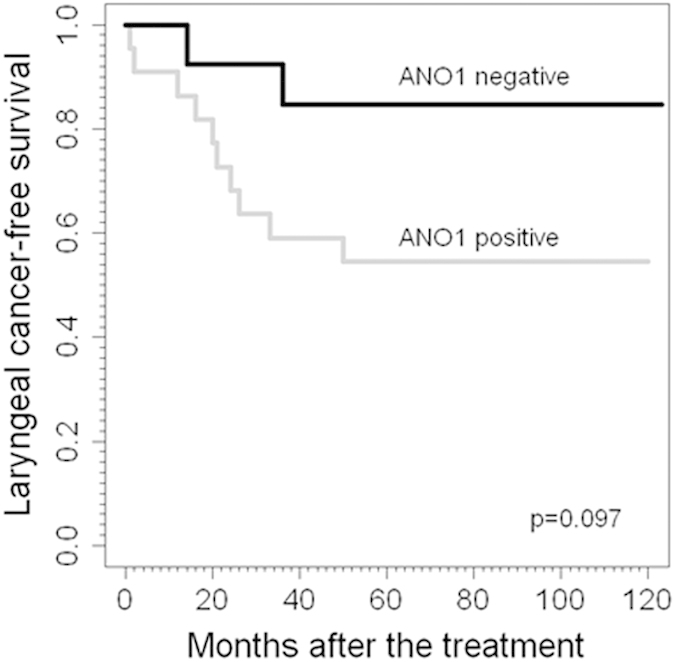
Cancer-free survival curves categorized by *ANO1* gene amplification (positive *versus* negative) in patients with laryngeal premalignancies.

We next studied the relationship of ANO1 protein expression and gene amplification with the risk of developing laryngeal cancer in patients with premalignant lesions. Interestingly, we found that ANO1 expression and, particularly, *ANO1* gene amplification associated with a higher frequency of progression to malignancy (Table 4). Consistent with these results, patients carrying lesions with *ANO1* gene amplification experienced a higher risk of cancer development (Fig. 3; HR = 3.62; 95% CI 0.79–16.57; *P* = 0.097, Cox regression), compared to patients carrying ANO1-positive lesions (HR = 1.65; 95% CI 0.44–6.24; *P* = 0.46, Cox regression).

## Discussion

11q13 amplification is a prevalent genetic alteration in HNSCC, observed in patients with advanced disease, a poorly differentiated histology of the tumour, and deeply invasive growth^3^. In concordance with the presumed association with progressed disease, the cases with amplification develop more frequently recurrences and have an increased risk of tumour-associated death^3^,^8^. It has been recently demonstrated that ANO1 expression and gene amplification significantly correlated with increased propensity to develop distant metastasis in HNSCC patients^6^. ANO1 has been implicated in various processes relevant to tumour progression and metastasis such as the regulation of cell migration, adhesion and invasion^6^,^7^,^9^,^10^,^11^. On the light of these data, *ANO1* gene emerges as a strong candidate to play a role in driving 11q13 amplification.

Nevertheless, current available information is very limited and controversial regarding the clinical relevance of ANO1 in HNSCC patients. Duvvuri *et al.*^12^ reported an association of ANO1 expression with decreased survival in HNSCC patients, although using a small sample size n = 34 and non-commercial antibodies, detecting ANO1 overexpression in 80% cases. Another study by Ruiz *et al.*^7^ reported ANO1 expression in 8% (19/242 cases) ranging 4–19% depending on the HNSCC site. Despite this low frequency, authors were able to find a correlation of ANO1 with poor prognosis, but it is worth noting that patients enrolled were subjected to different treatment regimens.

This prompted us to perform a comprehensive analysis of ANO1 expression and gene amplification using a large cohort of 372 homogeneously treated HNSCC patients. We detected ANO1 expression in 78 (22%) tumours, whilst *ANO1* gene amplification occurred at a higher frequency (58%). A positive correlation was found between ANO1 protein expression and gene amplification (*P* = 0.003). All ANO1-positive tumours harboured *ANO1* gene amplification; however, only one-third of cases with gene amplification were concomitantly accompanied by ANO1 overexpression. These results are in good agreement to those reported by Ruiz *et al.*^7^. However, we did not find an association of ANO1 expression with poor disease-specific survival, as previously reported. Various factors could contribute to these contrasting findings, such as differences in the inclusion criteria (e. g. patients undergoing different treatments, pooled HNSCC sites), sample sizes, detection methods and/or antibodies used.

Strikingly, we found important differences on the impact of ANO1 in patient survival depending on the anatomic site of the tumours. Thus, patients with ANO1-positive oropharyngeal tumours exhibited a significantly improved disease-specific survival, compared to hypopharyngeal and laryngeal tumours. These findings were confirmed by repeating IHC analysis using two different anti-ANO1 antibodies. HPV status data were available for the whole series. Given the low incidence of HPV in our series (3%, 10 HPV-positive cases), we can rule out the possible contribution of HPV infection as a confounding factor. A recent paper demonstrated that ANO1 expression in HNSCC is epigenetically regulated via promoter methylation, and interestingly, ANO1 was proposed as a primary driver of the “grow” or “go” model for HNSCC progression^13^. Thus, increased ANO1 expression may promote tumour growth, whereas decreasing ANO1 expression favours transition from an epithelial to a mesenchymal phenotype and the development of metastasis. This could explain the overall better prognosis observed in ANO1-positive patients in our series. Therefore, ANO1 emerges as a pleiotropic effector that seems to influence tumour proliferation and metastasis in an opposing way, which likely involves regulation through changes in phosphorylation and direct protein-protein interactions such as TMEM16A S970 and Radixin^13^. It has also been recently demonstrated that ANO1 interacts with EGFR and facilitates EGFR-signalling in HNSCC, thus revealing ANO1 as a promising target and/or biomarker for EGFR-directed therapy in HNSCC^14^.

Even though the role of ANO1 expression and gene amplification in tumour progression and metastatic dissemination has been investigated in different cancers, including HNSCC, its potential contribution to malignant transformation has not yet been explored in clinical samples. In an attempt to accomplish this, ANO1 protein expression and gene amplification were analysed on a set of laryngeal precancerous lesions, and molecular data correlated with the risk of progression to invasive carcinoma. We observed important differences when comparing the timing and frequency of both ANO1 protein expression and gene amplification in the different stages of HNSCC tumourigenesis. *ANO1* gene amplification was detected very early (in mild dysplasias) and at a high frequency that was maintained along progression; however, ANO1 protein expression was only found in severe dysplasias/CIS and at a lower frequency. More importantly, *ANO1* gene amplification but not ANO1 expression correlated almost significantly with increased laryngeal cancer risk.

In line with this, we described in a previous report that amplifications of *CCND1* and *CTTN* strongly and significantly predict laryngeal cancer development^14^. Both genes are in close proximity to *ANO1* within 11q13 amplicon. However, we found that CTTN (cortactin) expression, but not CCND1 (cyclin D1) expression, significantly predicted cancer risk in both larynx and oral cavity^15^,^16^.

We also previously demonstrated that *CTTN* gene amplification and protein overexpression correlated with poor prognosis and reduced patient survival in two large prospective series of HNSCC patients^4^,^17^, thus reinforcing the central role of *CTTN* in the 11q13 amplicon and also in disease progression. Hence, various genes are frequent targets of amplification within the 11q13 region that could cooperatively contribute to cancer development and progression.

In summary, our findings demonstrate that *ANO1* gene amplification occurs frequently in both premalignant lesions and invasive tumours, whereas concomitant ANO1 expression was detected at a much lower frequency. This study provides original evidence demonstrating the potential utility of *ANO1* gene amplification as a cancer risk marker in patients with laryngeal dysplasia, and also that the prognostic significance of ANO1 expression in HNSCC seems to be site-dependent.

## Methods

### Patients and Tissue Specimens

Surgical tissue specimens from 372 patients with HNSCC who underwent resection of their tumours at the Hospital Universitario Central de Asturias between 1990 and 2009 were retrospectively collected, in accordance to approved institutional review board guidelines. All experimental protocols were approved by the Institutional Ethics Committee of the Hospital Universitario Central de Asturias. Informed consent was obtained from all patients. Representative tissue sections were obtained from archival, paraffin-embedded blocks and the histological diagnosis was confirmed by an experienced pathologist (A.A.).

The sections were selected for study as follows: In premalignant lesions, the entire lesion was included in one block and therefore the section used for histological diagnosis was subsequently used for DNA/protein analysis. In carcinomas, three morphologically representative areas were selected from each individual tumour paraffin block for the construction of a tissue microarray (TMA), as described previously^18^.

The premalignant lesions were classified into categories of mild, moderate or severe dysplasia following the WHO classification^19^. Four (12%) lesions were classified as showing mild dysplasia, 12 (34%) as moderate dysplasia, and 19 (54%) as severe dysplasia/carcinoma *in situ* (CIS). All patients with premalignant lesions were men, with a mean age of 66 years (range 43–82 years). All of them were smokers and 23 were also habitual alcohol drinkers. Patients with a diagnosis of premalignant lesion and cancer within the next 6 months were excluded from the study. All patients were treated by excisional biopsy using stripping microflap excision with cold instruments. A complete macroscopic exeresis of the lesion was performed in all cases, but the microscopic margins were not addressed. Patients were followed-up for a minimum of 60 months or until progression to malignancy occurred.

A large unbiased cohort of 372 homogeneous surgically treated HNSCC patients was selected for study. All patients had a single primary tumour, microscopically clear surgical margins and received no treatment prior to surgery. Only fourteen patients were women, and the mean age was 59 years (range 30 to 86 years). All but twelve patients were habitual tobacco smokers, 198 moderate (1–50 pack-year) and 153 heavy (>50 pack-year), and 341 were alcohol drinkers. The stage of the tumours was determined according to the TNM system of the International Union Against Cancer (7th Edition): 18 tumours were stage I, 24 stage II, 64 stage III, and 266 stage IV. The series included 147 well, 145 moderately and 79 poorly differentiated tumours, determined according to the degree of differentiation of the tumour (Broders’ classification). 232 (62%) of 372 patients received postoperative radiotherapy.

### Tissue microarray (TMA) construction and DNA extraction

Five morphologically representative areas were selected from each individual tumour paraffin block: two for DNA isolation and three for the construction of a TMA. To avoid cross-contamination, two punches of 2 mm diameter were taken first, using a new, sterile punch (Kai Europe GmbH, Solingen, Germany) for every tissue block, and stored in eppendorf tubes at room temperature. Subsequently, three 1 mm cylinders were taken to construct TMA blocks, as described previously^18^,^20^, containing a total of 372 HNSCC (140 tonsillar, 108 base of tongue, 62 hypopharyngeal and 62 laryngeal carcinomas). In addition, each TMA included three cores of normal epithelium as an internal negative control. The protocol for DNA extraction from paraffin-embedded tissue sections has been described elsewhere^9^.

### Gene Amplification Analysis

Gene amplification was evaluated by Real-time PCR (Q-PCR) in an ABI PRISM 7500 Sequence detector (Applied Biosystems, Foster City, CA) using Power SYBR Green PCR Master Mix and oligonucleotides designed according to Primer Express software v2.0 with the following sequences: for *ANO1* gene, Fw, 5′- CAAAGGCAGGTGCTTTGCA -3′ and Rv, 5′- TCTACGGGCCTCTGCTCACT -3′, and for the reference gene *TH (Tyrosine Hydroxylase*, located at 11p15), Fw, 5′-TGAGATTCGGGCCTTCGA-3′ and Rv, 5′-GACACGAAGTAGACTGACTGGTACGT-3′. Dissociation curve analysis of all PCR products showed a single sharp peak and the correct size of each amplified product was confirmed by agarose gel electrophoresis. The relative gene copy number for *ANO1* was calculated using the 2^−ΔΔ*C*T^ method. The ΔΔ*C*_T_ represents the difference between the paired tissue samples (Δ*C*_T_ of tumour − Δ*C*_T_ of normal mucosa), with Δ*C*_T_ being the average *C*_T_ for the target gene (*ANO1*) minus the average *C*_T_ for the reference gene (*TH*). Values higher than 2.0 were considered positive for gene amplification.

### Immunohistochemistry

The formalin-fixed, paraffin-embedded tissues were cut into 3-μm sections and dried on Flex IHC microscope slides (Dako). The sections were deparaffinized with standard xylene and hydrated through graded alcohols into water. Antigen retrieval was performed using Envision Flex Target Retrieval solution, high pH (Dako). Staining was done at room temperature on an automatic staining workstation (Dako Autostainer Plus) with rabbit polyclonal Anti-TMEM16A antibody (Abcam #ab53212) at 1:500 dilution or with rabbit monoclonal Anti-TMEM16A [SP31] antibody (Abcam #ab64085) at 1:100 dilution using the Dako EnVision Flex + Visualization System (Dako Autostainer). Counterstaining with haematoxylin for 1 minute was the final step.

A semiquantitative scoring system based on staining intensity was applied. Immunostaining was scored blinded to clinical data by two independent observers as negative (0), weak to moderately (1+), and strongly positive (2+) based on staining intensity. Scores ≥ 1 were considered as ANO1 positive expression. Cut-off point was determined by survival.

### Statistical Analysis

Fisher’s exact test was used for comparison between categorical variables and odds ratios were reported as effect measure. Correlation between gene amplification and protein expression was tested by Spearman’s correlation coefficient. Due to the presence of competing risk events, the disease-specific survival was estimated using the multiple decrement method while the standard Kaplan-Meier was used for overall survival. Differences between curves were tested from the log-rank method. Hazard ratios and respective 95% confidence intervals, computed from the usual proportional hazard Cox model were reported as measure of the effect. All tests were two-sided. *P* values of ≤ 0.05 were considered to be statistically significant.

## Additional Information

**How to cite this article**: Rodrigo, J. P. *et al.* Clinical significance of *Anoctamin-1* gene at 11q13 in the development and progression of head and neck squamous cell carcinomas. *Sci. Rep.* **5**, 15698; doi: 10.1038/srep15698 (2015).

## Supplementary Material

Supplementary Information

## Figures and Tables

**Table 1:** Associations between ANO1 protein expression and clinicopathological findings in HNSCC patients (N = 357).

Characteristic	No.	ANO1-positive expression (%)	OR (95% CI)	P^#^
**- pT classification**				
T1-T2	107	28 (26)	1	0.169
T3	122	20 (16)	0.553 (0.290–1.054)	
T4	125	30 (24)	0.891 (0.491–1.616)	
**- pN classification**				
N0	93	18 (19)	1	0.499
N1-3	264	60 (23)	1.225 (0.680–2.210)	
- Disease stage				
I–II	37	11 (30)	1	0.108
III	63	8 (13)	0.344 (0.124–0.957)	
IV	257	59 (23)	0.704 (0.329–1.510)	
**- Pathological grade**				
Well differentiated	140	35 (25)	1	0.117
Moderately differentiated	141	23 (16)	0.585 (0.325–1.053)	
Poorly differentiated	75	20 (27)	1.091 (0.576–2.067)	
**- Site**				
Oropharynx	240	60 (25)	1	0.065
Hypopharynx	60	12 (20)	0.750 (0.374–1.505)	
Larynx	57	6 (11)	0.353 (0.144–0.864)	
**Total Cases**	357	78 (22)		

#*P* value associated to the odds ratio (OR).

**Table 2:** Five-year disease-specific survival (DSS) and overall survival (OS) according to ANO1 expression.

Patients	ANO1-positive expression	ANO1-negative expression	HR (95% CI); *P*
Whole series:			
- DSS	56%	44%	0.76 (0.53- 1.08); 0.13
- OS	47%	36%	0.75 (0.55- 1.02); 0.07
Oropharyngeal tumours			
- DSS	61%	44%	0.63 (0.41–0.98); 0.04
- OS	49%	35%	0.68 (0.47–0.98); 0.04
Hypopharyngeal tumours			
- DSS	51%	36%	0.77 (0.36–1.67); 0.51
- OS	37%	28%	0.67 (0.31–1.42); 0.3
Laryngeal tumours			
- DSS	63%	51%	1.62 (0.56–4.7); 0.38
- OS	52%	43%	1.35 (0.47–3.8); 0.58

HR: hazard ratio; CI: Confidence interval.

**Table 3:** ANO1 protein expression and gene amplification in relation to histopathologic diagnosis in laryngeal premalignant lesions.

Diagnosis	No. patients (%)	*ANO1 gene* amplification (%)	ANO1 protein Expression (%)
Mild Dysplasia	4 (12)	3 (75)	0
Moderate Dysplasia	12 (34)	6 (50)	0
Severe Dysplasia	19 (54)	13 (68)	7 (37)
Total	35	22 (63)	7 (20)

**Table 4:** Evolution of the premalignant lesions in relation to histopathologic diagnosis, *ANO1* gene amplification and protein expression at five years.

Characteristic	No of cases (%)	Progression to carcinoma (%)	*P*†
Histopathologic diagnosis			
Mild dysplasia	4 (12)	0 (0)	0.117
Moderate dysplasia	12 (34)	2 (17)	
Severe dysplasia	19 (54)	9 (47)	
*ANO1* gene amplification			
Negative	13 (37)	2 (15)	0.139
Positive	22 (63)	10 (45)	
ANO1 protein expression			
Negative	28 (80)	8 (29)	0.652
Positive	7 (20)	3 (43)	

^†^Fisher’s exact test.
