# Supplementary material for: Clinical significance of Anoctamin-1 gene at 11q13 in the development and progression of head and neck squamous cell carcinomas
Source: Sci Rep. 2015 Oct 26;5:15698. doi: 10.1038/srep15698 (PMC4620505; doi:10.1038/srep15698)
Supplement: Supplementary Information [file srep15698-s1.pdf]

Supplementary Information:

CLINICAL SIGNIFICANCE OF *ANOCTAMIN-1* GENE AT 11q13 IN THE DEVELOPMENT AND PROGRESSION OF HEAD AND NECK SQUAMOUS CELL CARCINOMAS

Juan P. Rodrigo[1]*‡, Sofía Tirados Menéndez[1]‡, Francisco Hermida-Prado[1], Saúl Álvarez-Teijeiro[1], María Ángeles Villaronga[1], Laura Alonso-Durán[1], Aitana Vallina[2], Pablo Martínez-Camblor[3,4], Aurora Astudillo[2], Carlos Suárez[1], Juana María García-Pedrero[1].

[1] Servicio de Otorrinolaringología, Hospital Universitario Central de Asturias and Instituto Universitario de Oncología del Principado de Asturias, Oviedo, Spain.

[2] Servicio de Anatomía Patológica, Hospital Universitario Central de Asturias and Instituto Universitario de Oncología del Principado de Asturias, Oviedo, Spain.

[3] Bioestadística, Hospital Universitario Central de Asturias, Oviedo, Asturias, Spain.

[4] Universidad Autónoma de Chile, Santiago, Chile.

‡These authors contributed equally to this work.

Supplementary Table S1: Five-year disease-specific survival (DSS) and overall survival (OS) according to ANO1 expression using [SP31] antibody.

**Patients**	**ANO1-positive expression**	**ANO1-negative expression**	**HR (95% CI); *P***
**Whole series:**			
- DSS	55%	44%	0.79 (0.55- 1.12); 0.19
- OS	44%	36%	0.78 (0.57- 1.06); 0.14
**Oropharyngeal tumours**			
- DSS	63%	42%	0.64 (0.41-1.0); 0.05
- OS	47%	33%	0.67 (0.46-0.98); 0.03
**Hypopharyngeal tumours**			
- DSS	34%	32%	0.86 (0.43-1.7); 0.66
- OS	30%	29%	0.84 (0.44-1.62); 0.6
**Laryngeal tumours**			
- DSS	62%	50%	1.27 (0.38-4.27); 0.7
- OS	53%	50%	1.05 (0.32-3.5); 0.92

HR: hazard ratio; CI: Confidence interval
